# Rare Source of Embolism in a Young Patient: Case Report and Literature Review

**DOI:** 10.3390/jcm11072038

**Published:** 2022-04-05

**Authors:** Marianna Vachalcová, Monika Jankajová, Marta Jakubová, Karolina Angela Sieradzka, Tibor Porubän, Gabriel Valočik, Peter Šafár, Daniela Ondušová, Ján Petruš, Ingrid Schusterová

**Affiliations:** 1 1st Department of Cardiology, East-Slovak Institute of Cardiovascular Diseases, 04011 Kosice, Slovakia; marianna.vachalcova@gmail.com (M.V.); sieradzka.ina@gmail.com (K.A.S.); porubantibor@gmail.com (T.P.); gvalocik@vusch.sk (G.V.); jpetrus@vusch.sk (J.P.); 2 Department of Functional Diagnostic, East-Slovak Institute of Cardiovascular Diseases, 04011 Kosice, Slovakia; mjakubova@vusch.sk (M.J.); dondusova@vusch.sk (D.O.); ischusterova@vusch.sk (I.S.); 3 Department of Cardiothoracic Surgery, East-Slovak Institute of Cardiovascular Diseases, 04011 Kosice, Slovakia; psafar@vusch.sk

**Keywords:** myocardial infarction, thrombus, thrombophilia, embolism

## Abstract

We present a case of a 31-year-old patient, smoker, with no previous medical history, presenting with acute limb ischemia and infarction of the spleen due to peripheral embolism. The source of embolism was thrombi formations in the left ventricular cavity, located in the area of the regional wall motions abnormalities. CT and coronary angiography confirmed the total occlusion of the left anterior descending artery with collateralization. The patient underwent acute bilateral embolectomy of the iliac, femoral, and popliteal arteries. Subsequently, cardiothoracic surgery was indicated with coronary bypass surgery and extirpation of left ventricular masses, later confirmed as thrombus by pathology characteristics. Hematological examinations proved homozygous thrombophilia, and the patient was indicated for lifelong anticoagulation therapy.

## 1. Introduction

Myocardial infarction (MI) with thrombus formation in the left ventricular cavity is a rare source of embolism in a young patient. We describe the unique case of a young patient presenting with acute limb ischemia and spleen infarction due to peripheral embolism of a thrombus after MI.

## 2. Case Report

We report a case of a 31-year-old man, smoker, with no previous medical history, with negative family history of thrombotic and hemorrhagic events, who, in September 2021, was referred to the emergency department for pain and reduced sensitivity in his lower limbs. The pain appeared a month earlier and was preceded by chest pain radiating to his neck and arms. Angiological examination confirmed acute limb ischemia. Ultrasound and CT angiography showed subtotal occlusion in the distal part of bilateral femoral and popliteal arteries, subtotal occlusion of the superior mesenteric artery, infarction of the spleen, and post-infarction change in the kidneys. Blood pressure was 127/92 mmHg. An ECG showed sinus rhythm, 2 mm ST-segment elevations in leads V2–V3, and Q wave and negativization of T wave in leads V1–V5. Troponin I levels were elevated (374 ng/L), NT-pro-B-type natriuretic peptide was 8500 ng/L, and creatine kinase and myoglobin peaked at 92 ukat/L and 17,107 ug/L, respectively. The complete blood count was normal. The patient had a low-risk profile for atherosclerosis coronary artery disease.

**Figure 1:**
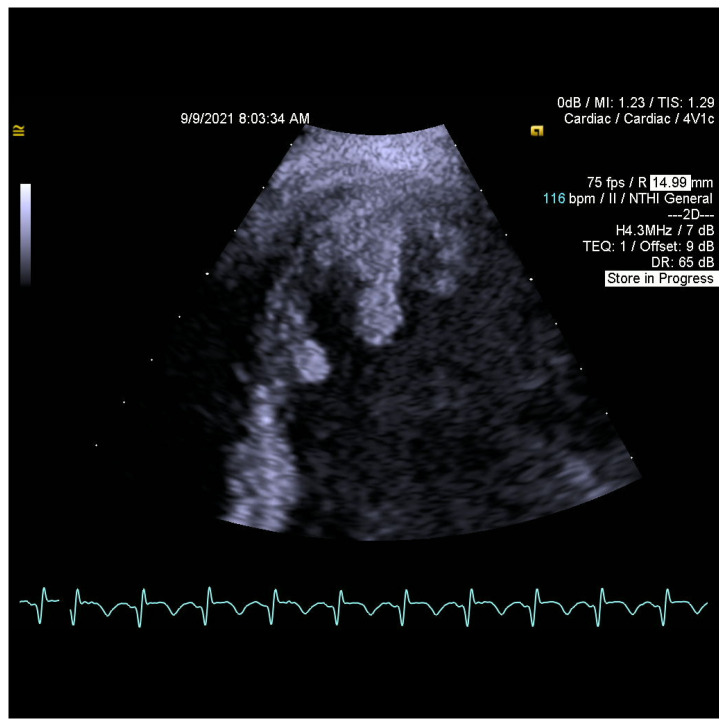
Thrombi in the left ventricular apex with high embolic potential.

**Figure 2:**
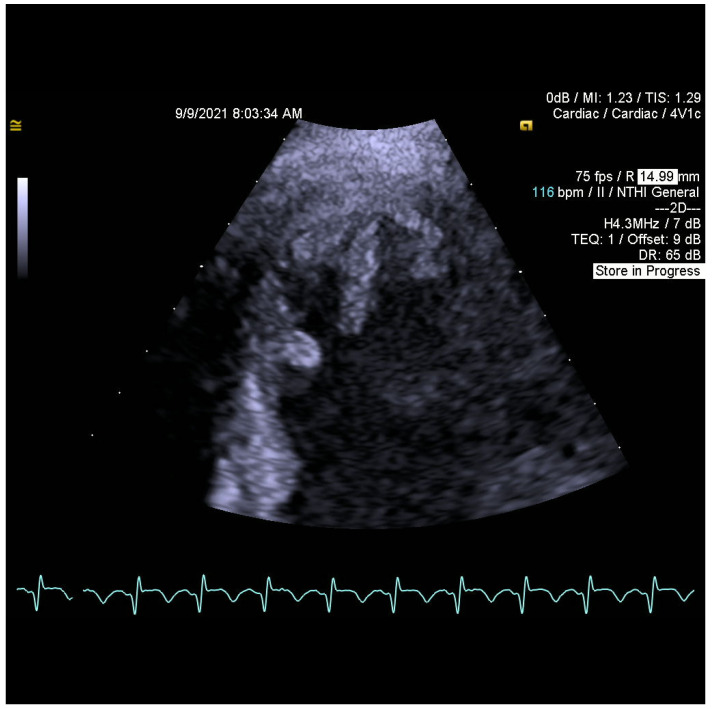
Another focus on thrombi in the left ventricular apex by TTE.

When searching for a source of embolization, transthoracic and transesophageal echocardiograms (TTE and TOE) were performed. They revealed flowing structures in the left ventricular apex with high embolic potential (Figure 1 and Figure 2), reduced left ventricular ejection fraction (EF LV 38%), akinesis of the left ventricular apex, hypokinesis of adjacent segments of the lateral wall, interventricular septum, anterior and inferior wall, and no significant valvular disease.

**Figure 3:**
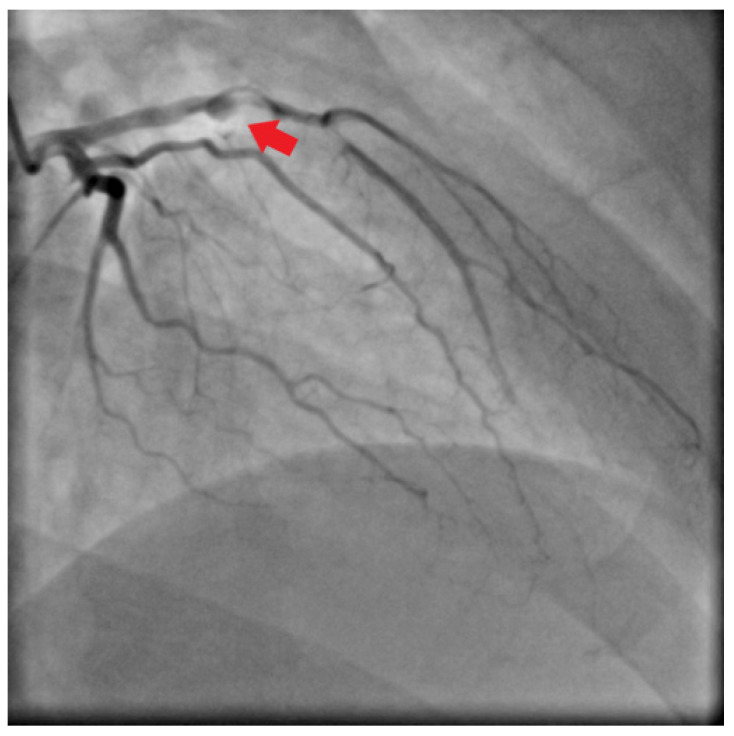
Selective coronary angiography showed total occlusion of left anterior descending artery with collateralization (red arrow).

Emergent surgical revascularization was indicated, and the patient underwent bilateral embolectomy of iliac, femoral, and popliteal arteries. Additional CT angiography and subsequently selective coronary angiography showed total occlusion of the left anterior descending artery with collateralization (Figure 3).

**Figure 4:**
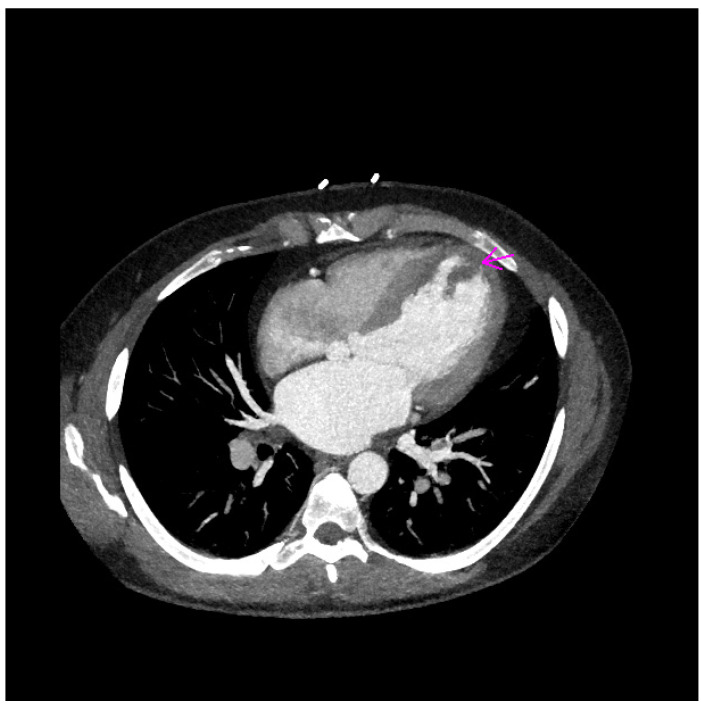
CT finding of first thrombus in the area of left ventricular apex (purple arrow).

**Figure 5:**
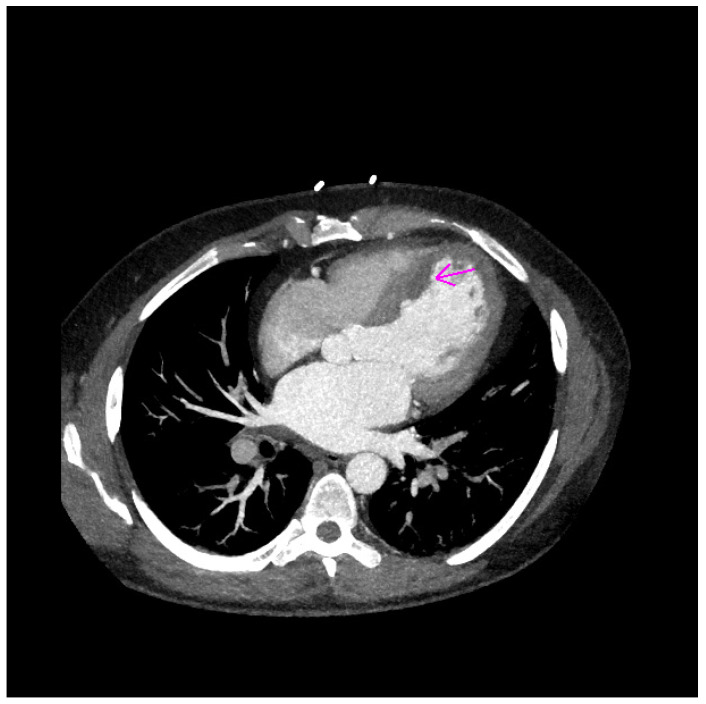
CT finding of second thrombus in the area of interventricular septum (purple arrow).

CT examination confirmed two floating structures (20 × 16 × 8 mm and 27 × 11 × 15 mm) in the area of the interventricular septum and left ventricular apex (Figure 4 and Figure 5).

Cardiothoracic surgery was indicated by the Heart Team. Coronary artery bypass grafting (left internal mammary artery was used for revascularization of the left anterior descending artery) and removal of left ventricular masses were performed. Pathology characteristics confirmed the thrombus. Therefore, hematological screening was supplemented. The examinations positively proved antiphospholipid syndrome. The screening assay APTT–LA was used, and extended APTT was detected. The lupus anticoagulants testing was positive and repeated in 12 weeks with persistence of the antibody. Laboratory testing for anticardiolipin antibodies and anti-β2-glycoprotein I antibodies was not performed. Moreover, genetic testing assessed 4G/4G homozygosity for the PAI-1 gene. The control TTE revealed persistent regional wall motion abnormalities, reduced LV EF (38%), and no significant valvular diseases. The treatment of heart failure was boosted, and lifelong anticoagulation therapy was indicated. At the 5-month follow-up, there was no evidence of embolic recurrence.

## 3. Discussion

Thrombophilia is a common risk factor for venous thromboembolism. Arterial thrombosis and the risk of MI, particularly among young people with low atherosclerotic burden, are associated mainly with the presence of antiphospholipid syndrome. [1] In young patients with MI and conventional risk factors, the probability of thrombophilia is high. Thrombophilia may lead to MI in this specific group of patients. [2] The left anterior descending artery is more frequently the culprit artery of MI in young patients. [3] Previous studies have reported that atrial fibrillation is the underlying disease in patients with coronary embolism (CE). CE has also been detected in the case of concomitant cardiomyopathy, valvular heart disease, infective endocarditis, antiphospholipid antibody syndrome, autoimmune disorders, and malignancy. A total of 26.4% of cases were of unclear etiology [4]. CE is diagnosed with coronary angiography, concomitant CE in multiple locations, or evidence of systemic embolization. The source of embolism should be revealed. An important step is the identification of a thrombus or intracardiac shunt using echocardiography. CE is frequently associated with systemic embolism, which should be screened [5]. MI is caused by CE in 4% to 13% of cases [4,6]. Paradoxical embolism should also be considered in the presence of thrombophilia and a hypercoagulable risk factor [7,8,9].

We present a unique case of a young patient without previous medical history who overcame MI. The known risk factor was smoking. The first symptoms of MI and peripheral embolization appeared a month before hospitalization. We suppose that due to the total occlusion of the left anterior descending artery, the thrombi with high embolic potential were formed in the area of regional wall motions abnormalities. The patient was admitted to hospital presenting with acute limb ischemia. The source of embolism was with high probability the thrombi in the left ventricle. The cardiosurgical operation and removal of the floating thrombi, later confirmed by pathology characteristics, was indicated. Hematological screening revealed positivity of antiphospholipid syndrome, and genetic testing assessed 4G/4G homozygosity for the PAI-1 gene. Thrombectomy, sometimes aided by intracoronary glycoprotein IIb/ IIIa inhibitors or thrombolytic therapy, is performed if the diagnosis of CE is established during coronary angiography. Coronary stents are not commonly required. Oral anticoagulation therapy needs to be initiated. A search for risk factors needs to be conducted. There is no need for routine thrombophilia screening unless there is clinical suspicion [5].

In the presented case report, we suppose the CE related to thrombophilia and peripheral embolism was due to the thrombi formation in the left ventricle. According to the history of chest pain one month before hospitalization, overcomed MI was diagnosed. The culprit coronary artery, the left anterior descending artery, was in accordance with previous trials. The patient was indicated for lifelong anticoagulation therapy. The treatment of heart failure was also initiated.

In young patients with MI, routine thrombophilia screening is indicated in the case of clinical suspicion; that was also the case with our patient.

## 4. Conclusions

The main goal of this case report is to highlight the diagnostic approach in young patients with arterial thromboembolism. Up-to-date, routine thrombophilia screening is indicated in the case of clinical suspicion and should be considered in young patients presenting with myocardial infarction and peripheral embolism.

## References

[1] Boekholdt S.M., Kramer M.H. (2007). Arterial thrombosis and the role of thrombophilia. Semin. Thromb. Hemost..

[2] Segev A., Ellis M.H., Segev F., Friedman Z., Reshef T., Sparkes J.D., Tetro J., Pauzner H., David D. (2005). High prevalence of thrombophilia among young patients with myocardial infarction and few conventional risk factors. Int. J. Cardiol..

[3] Zasada W., Bobrowska B., Plens K., Dziewierz A., Siudak Z., Surdacki A., Dudek D., Bartuś S. (2021). Acute myocardial infarction in young patients. Kardiol. Pol..

[4] Shibata T., Kawakami S., Noguchi T., Tanaka T., Asaumi Y., Kanaya T., Nagai T., Nakao K., Fujino M., Nagatsuka K. (2015). Prevalence, clinical features, and prognosis of acute myocardial infarction attributable to coronary artery embolism. Circulation.

[5] Gulati R., Behfar A., Narula J., Kanwar A., Lerman A., Cooper L., Singh M. (2020). Acute Myocardial Infarction in Young Individuals. Mayo Clin. Proc..

[6] Prizel K.R., Hutchins G.M., Bulkley B.H. (1978). Coronary artery embolism and myocardial infarction. Ann. Intern. Med..

[7] Kanwar S.M., Noheria A., DeSimone C.V., Rabinstein A.A., Asirvatham S.J. (2016). Coincidental impact of transcatheter patent foramen ovale closure on migraine with and without aura—A comprehensive meta-analysis. Clin. Trials Regul. Sci. Cardiol..

[8] Kleber F.X., Hauschild T., Schulz A., Winkelmann A., Bruch L. (2017). Epidemiology of myocardial infarction caused by presumed paradoxical embolism via a patent foramen ovale. Circ. J..

[9] Chen W., Yu Z., Li S., Wagatsuma K., Du B., Yang P. (2021). Concomitant acute myocardial infarction and acute pulmonary embolism caused by paradoxical embolism: A case report. BMC Cardiovasc. Disord..

